# Etymologia: *Anaplasma phagocytophilum*

**DOI:** 10.3201/eid2504.ET2504

**Published:** 2019-04

**Authors:** Ronnie Henry

**Keywords:** *Anaplasma phagocytophilum*, bacteria, human granulocytic anaplasmosis, vectorborne infections, tickborne infections, human granulocytic ehrlichiosis

## *Anaplasma phagocytophilum* [anʺǝ-plazʹmǝ faʹgo-sītʺo-fī-lum]

A species of tickborne bacteria that causes human granulocytic anaplasmosis, *Anaplasma* (from the Greek *an*- [“without”] + *plasma* [“shape”]) *phagocytophilum* (named for its affinity for growing in neutrophils: phagocyte + Latin *phile* [“loving”]) has gone by many names (Figure). First it was named *Rickettsia* (for Howard Taylor Ricketts) *phagocytophilum*, then *Cytoecetes* (for its similarity to *Cytoecetes microti*) *phagocytophilum*, and then *Ehrlichia* (for Paul Ehrlich) *phagocytophilum*. More recently, *E. equi* and the agent of human granulocytic ehrlichiosis (now anaplasmosis) were combined with *E. phagocytophilum* as *A. phagocytophilum*.

**Figure:**
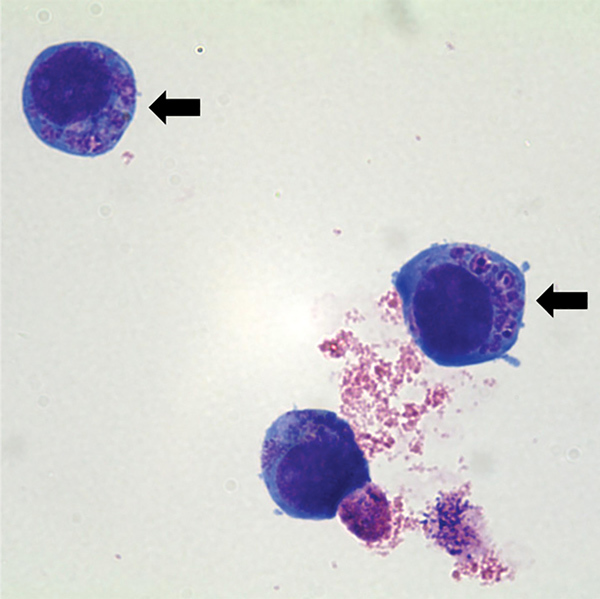
*Anaplasma phagocytophilum* cultured in human promyelocytic cells, showing morulae as basophilic and intracytoplasmic inclusions (arrows). Wright-Giemsa stain. Original magnification x1,000. Image: Emerg Infect Dis. 2014;20:1708–11.
